# The influence of the interaction between platform types and consumer types on the purchase intention of live streaming

**DOI:** 10.3389/fpsyg.2022.1056230

**Published:** 2022-11-28

**Authors:** Ying Xie, Kai Du, Peng Gao

**Affiliations:** School of Economics and Management, Northwest University, Xi’an, China

**Keywords:** platform types, consumer types, rational thinking, emotional thinking, live streaming shopping

## Abstract

Under the background of the rapid development of live streaming shopping industry, diversified live streaming platforms have emerged one after another. This study aims to explore the interaction effect of platform types (live streaming embedded in e-commerce platforms/LSEEC vs. e-commerce integrated into live streaming platforms/ECILS) and consumer types (goal-driven consumers vs. recreational consumers) on purchase intention. To verify the effect, this study firstly conducted a laboratory experiment (Study 1), and then carried out a questionnaire survey through the Internet (Study 2). The results indicate that the interaction effect of platform types and consumer types exists (Study 1) and thinking patterns play a mediating role (Study 2): for goal-driven consumers, LSEEC platforms are more likely to stimulate their rational thinking and enhance their purchase intention; for recreational consumers, ECILS platforms are more likely to stimulate their emotional thinking and enhance their purchase intention. The findings expand the depth of research related to live streaming platforms, deepen the understanding of the thinking patterns in live streaming shopping decision-making, and have certain guiding significance for the strategic formulation of enterprises.

## Introduction

With the hot development of live streaming shopping, the competition between live shopping platforms has become increasingly fierce (Zhu and Zhu, 2022). The study found that in addition to traditional e-commerce platforms such as Taobao, JD and Mushroom Street, new entertainment platforms such as Tik Tok and Kwai have also opened the live shopping function (Zhao, 2018; Yang and Wang, 2021; Zhu and Zhu, 2022). The former’s traditional e-commerce platforms are exactly what the live streaming research calls “live streaming embedded e-commerce (LSEEC)” platforms, while the latter’s new entertainment platforms are “e-commerce integrated into live streaming (ECILS)” platforms (Cai et al., 2018). Data shows that these two types of platforms have their own characteristics (iiMedia Research, 2020a). LSEEC platforms: have more transaction attributes; are rich in categories; users on them have relatively clear shopping purpose; live conversion rate of these platforms is relatively high (Zhao, 2018). While the ECILS platforms: have more social attributes; are rich in the number of fans; user stickiness of these platforms is stronger; the channels of these platforms are more down. How to combine the characteristics of the platform to do a good job on product sales has become a key issue in marketing decisions. Therefore, it is crucial to explore the context, target groups and the mechanisms of the LSEEC platforms and the ECILS platforms. It will help companies to efficiently strategize live streaming platform marketing strategies. However, few live streaming studies have concerned different platform types and their target groups. Most of the existing studies are based on a single platform: one branch of research is based on LSEEC platforms and explores customer engagement (Hu and Chaudhry, 2020; Guo et al., 2021), consumer’ purchase intention (Sun et al., 2019; Chen et al., 2020; Liu et al., 2020; Rungruangjit, 2022), factors influencing celebrity live streaming (Luo et al., 2021) and publishing marketing strategies (Liu, 2020); the other branch of research is based on ECILS platforms and explores consumer’ purchase intention (Li et al., 2021; Ho et al., 2022; Zhang et al., 2022), user behavior prediction (Gao et al., 2022) and book live streaming (Wang and Zhang, 2021). So how to choose platforms to increase consumers’ purchase intentions when dealing with different types of consumers? What is its internal mechanism?

Unlike offline shopping and traditional online shopping, live shopping is a “live+shopping” marketing model that uses the live streaming function of the platform (Wang, 2021). This study argues that the type of live streaming platform affects consumers’ thinking patterns. According to the dual processing theory, there are two modes of cognitive thinking in consumption: rational thinking and emotional thinking (Strack et al., 2006). Rational thinking is a slow, controlled, deliberate, externally influenced, rule-based thinking process that requires the involvement of working memory and more cognitive effort; emotional thinking is a fast, spontaneous, associative, heuristic-based thinking process that requires less cognitive effort and emphasizes intuition and experience (Evans, 2008). LSEEC platforms focus on shopping transactions and offer a wide range of products with a high degree of professionalism, driving live viewers to purchase goods through rational thinking; ECILS platforms with stronger social and entertainment attributes are more attractive to viewers, driving them to purchase goods through emotional thinking. Besides, different customers may think and behave differently according to the characteristics of platforms: Goal-driven consumers watch live shopping just to complete their shopping plans. They place considerable value on the efficiency and affordability when they do shopping. So they are more likely to make their purchase decisions through rational thinking. Recreational consumers watch live shopping more for entertainment to spend their time, and the fun of live streaming is the key to attract them. Thus recreational consumers rely more on emotional thinking, such as intuition and experience, to make decisions in live streaming (Shen and Wang, 2002; Chen and Dong, 2017). Therefore, the question further explored in this study is whether there will be an interaction between the live streaming platform types and consumer types.

In order to examine the interaction effect, this paper conducts two studies. In Study 1, we perform an experiment to demonstrate that LSEEC platforms enhance the purchase intention of goal-driven consumers and ECILS platforms enhance the purchase intention of recreational consumers. To verify the external validity of Study 1 and explore the psychological mechanisms of the interaction effect, study 2 again demonstrates the interaction effect and verifies the mediating mechanism through a large-scale online questionnaire. The results show that goal-driven consumers are more likely to purchase on LSEEC platforms, where rational thinking plays a mediating role; recreational consumers are more likely to purchase on ECILS platforms, where emotional thinking plays a mediating role.

## Theoretical background and hypotheses

### Interaction between platform types and consumer types

Live streaming shopping is a new type of shopping that contains not only a large number of social commerce attributes, but also unique social media attributes. Research points out that platforms of live shopping can be divided into two categories: one is Live Streaming Embedded in E-Commerce (LSEEC), such as Taobao, JD, Amazon, etc.; the other is E-Commerce Integrated into Live Streaming (ECILS), such as Tik Tok, Kwai, etc. (Cai et al., 2018). On the one hand, the latest research finds that LSEEC platforms and ECILS platforms have different signaling attributes from the perspective of signaling theory: LSEEC platforms are considered to be focused on providing information about goods, while ECILS platforms are considered to be socially focused (Lu and Chen, 2021). On the other hand, data shows that the LSEEC platform is dominated by merchants (iiMedia Research, 2020b). Their product expertise can provide consumers with more accurate and comprehensive information, which can easily stimulate consumers’ information discernment (Erdogan, 1999) and help them make better purchase decisions. While the ECILS platform is dominated by online celebrities (iiMedia Research, 2020b), which can easily stimulate consumers’ emotional discernment and enhance consumers’ satisfaction and pleasure (Raghunathan and Corfman, 2006).

Research has found that in addition to objective factors such as product types (Huang and Wang, 2019) and shopping environment (Ma et al., 2012), consumers’ subjective factors also influence the final purchase decision (Zhu et al., 2004). According to the specific situation of online consumption in China and the standard of whether online shopping behavior is planned or not, some studies have divided consumers into two categories: recreational and goal-driven (Shen and Wang, 2002; Chen and Dong, 2017). This coincides with the classification of consumer purchase behavior in traditional marketing theory (Shen and Wang, 2002). It was found that recreational consumers are characterized by browsing shopping websites or watching live shopping when they have spare time to find out what is worth buying. They seek various pleasures through the purchase process, such as unexpected surprises, more involvement and symbolic meaning of the product. Thus these consumers tend to rely on intuition rather than intellect and deliberation when they make purchase decisions. Goal-driven consumers only visit shopping sites or watch live shopping when they have a shopping plan. They are task-oriented, efficient, sensible and cautious. They want to buy what they want quickly rather than for pleasure or entertainment (Chen and Dong, 2017). These consumers tend to view purchases as a form of “work” and often use words that describe work performance, such as “success” or “completion” to evaluate the results of their efforts (Shen and Wang, 2002).

The LSEEC platform is centered on shopping and providing professional information about products, and the platform’s ease of use and practical features facilitate consumers to do operations such as compare product prices and assess return losses. For example, the search function provided by the platform helps consumers to go straight to the live room of target brand products and similar brand products, which saves time costs and allows them to compare products; the platform’s post-purchase reviews show consumers the experiences of other users, which reduces decision risks and potential return losses, etc. (Hao, 2010). In addition, the live broadcast of LSEEC platforms is dominated by corporate anchors. They have a good understanding of product performance and parameters, which can meet consumers’ information needs in the purchase process and improve their decision-making efficiency. In contrast, the ECILS platform is dominated by internet celebrities, which limited the depth and breadth of the introduction because it is difficult for internet celebrities to give accurate, reliable, comprehensive and professional answers about the product. As a result, it is less likely to meet the information needs of consumers to make reasonable decisions (Huang et al., 2021). Therefore, compared to the ECILS platform, the LSEEC platform is better able to meet the needs of goal-driven consumers.

For the recreational consumer who has no definite shopping plan, the focus of shopping is fun, enjoyment and passing the time. The ECILS platform focuses more on social entertainment and advocates providing users with more fun and enjoyment. For example, the platforms’ business model of “content is king” and “social interaction is paramount” perfectly meets the needs of users (Wang and Jiang, 2021). The ECILS platform’s e-commerce model, which stimulates users’ consumption needs through content creation to achieve sales conversion, creates a relaxed and positive atmosphere that makes users feel fun (Zang and Zhao, 2021). In addition, the ECILS platform is mainly dominated by internet celebrities, who often have good appearance and unique personal charisma. The live streaming is usually full of charm, which is more likely to attract consumers and enhance consumers’ satisfaction and pleasure during viewing (Raghunathan and Corfman, 2006). These characteristics will lead to a greater allure of the product to consumers. In contrast, the LSEEC platform is relatively short in social entertainment elements. The pleasure and satisfaction it brings to consumers are insufficient to convincing recreational consumers. Therefore, the ECILS platform matches the needs of the recreational consumer better than the LSEEC platform.

Based on the above discussion, we argue that there is an interaction effect between platform types and consumer types, and propose the following hypothesis:

*H1*: There is an interactive effect of platform types and consumer types on purchase intention.

*H1a*: For goal-driven consumers, LSEEC platforms are more likely to enhance purchase intention compared to ECILS platforms.

*H1b*: For recreational consumers, ECILS platforms are more likely to enhance purchase intention than LSEEC platforms.

### Mediating role of cognitive thinking patterns

Dual Processing Theory (DPT) is one of the most influential theoretical models in the field of reasoning and decision making. The theory suggests that there are two types of cognitive thinking patterns: rational thinking and emotional thinking. Rational thinking is a slow, controlled, deliberate, externally influenced and rule-based thinking process that requires the involvement of working memory and more cognitive effort. Emotional thinking is intuitive, fast, spontaneous, associative, heuristic-based, requiring less cognitive effort and favoring intuitive thinking in people’s cognition (Evans, 2008). And compared with emotional thinking, rational thinking requires more attention to information when it plays a role (Zhang et al., 2014; Wang, 2015). At the same time, it has been found that cognitive thinking patterns are more inclined to a specific state, strongly influenced by specific situations (Novak et al., 2009) and individual factors (Sloman, 1996). For example, the study found that when consumers buy low-price products on the mobile phone, it is easy to stimulate emotional thinking. On the contrary, when consumers buy high-price products on the personal computer, it is easy to trigger rational thinking (Huang and Wang, 2019). Similarly, when consumption decisions are related to time rather than money, people tend to make decisions through emotional thinking, that is, they rely more on intuition and experience (Samson and Voyer, 2014; He et al., 2021). Meanwhile, the thinking patterns on which decision-making depends will also be different when consumers’ preferences are different (Del Missier et al., 2012).

Specifically in live shopping: the goal-driven consumers, as planned and efficiency-oriented consumers, are more result-oriented when doing shopping. With the goal of completing the plan, goal-driven consumers consider object factor more in shopping (Chen and Dong, 2017). The LSEEC platform focuses on shopping. It offers abundant product information and encompasses a variety of products, which can effectively meet the information needs for goal-driven consumers to purchase goods. At the same time, the platform also provides a variety of brands and similar products from multiple merchants, which makes it easy for consumers to compare the products, save shopping costs, and achieve economic benefits. In addition, the professional product information provided by the majority of corporate anchors on the LSEEC platform (Huang et al., 2021) provides a basis for consumer decisions, and better satisfies the needs of goal-driven users to solve problems through rational thinking than the ECILS platform. Therefore, this paper argues that LSEEC platforms can better stimulate the rational thinking of goal-driven consumers to make purchase decisions. And the recreational consumers, as impulsive, fun-focused and pleasure-oriented individuals (Chen and Dong, 2017), aim to seek out fun to pass the time. The ECILS platform stimulates consumers’ emotional thinking by pleasing them and reinforcing their emotions and attachment to the platform (Ma and Wang, 2015). The rich live streaming creation content of the platform is very attractive, satisfying the users’ leisure and entertainment. At the same time, the platform’s large number of internet celebrity anchors often have good appearance and unique personal charm (Huang et al., 2021), and their interaction with customers while selling goods reinforces users’ sense of engagement, satisfaction and pleasure. Thus, this paper argues that the ECILS platforms are more likely to stimulate the emotional thinking of recreational consumers and promote them to make purchase decisions.

*H2a*: On LSEEC platforms, goal-driven consumers’ rational thinking is easily inspired, which promotes them to make purchase decisions.

*H2b*: On ECILS platforms, recreational consumers’ emotional thinking is easily inspired, which promotes them to make purchase decisions.

**Figure 1:**
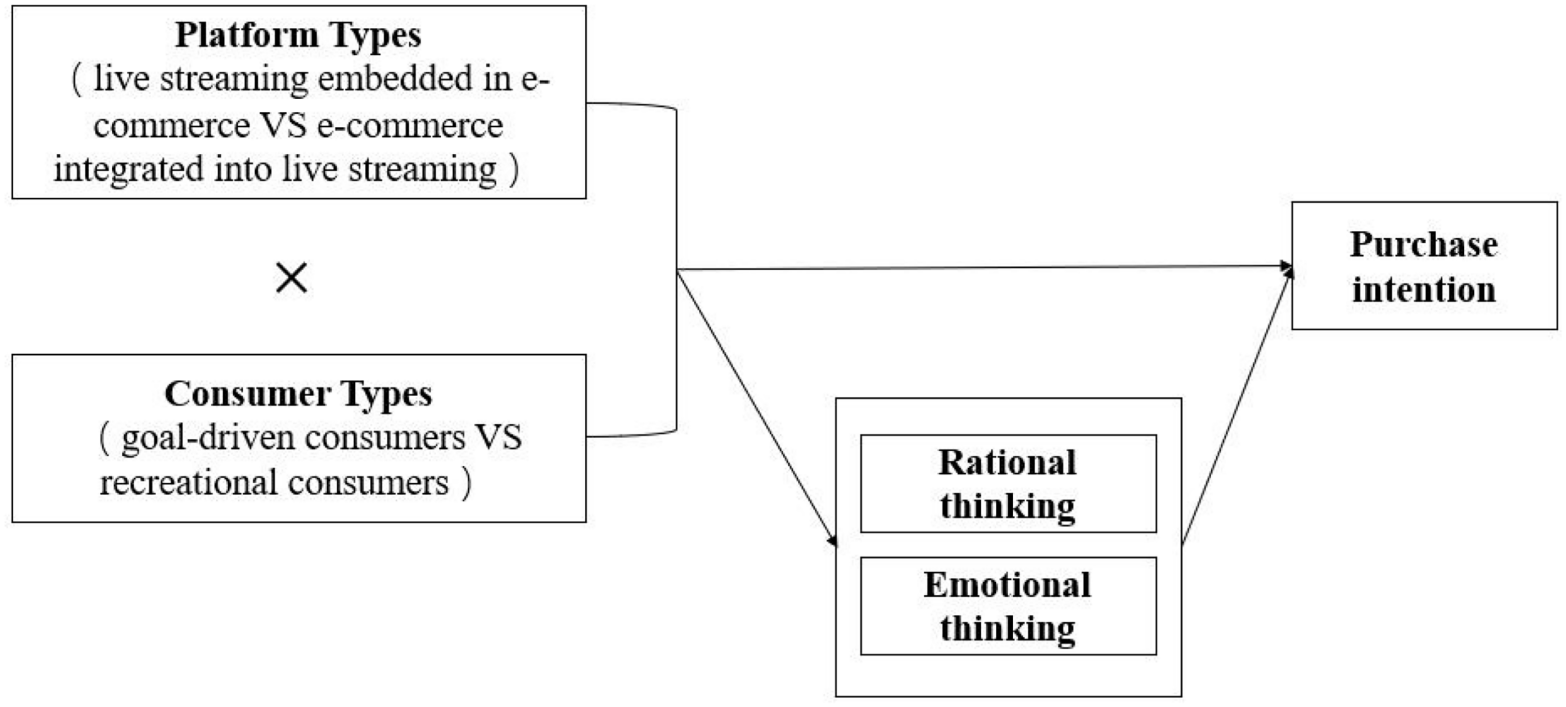
Theoretical model.

The theoretical model of this study is shown in Figure 1.

## Study 1: The interaction effect of platform types and consumer types

Study 1 explored the interaction between platform types and consumer types on purchase intention. Consistent with H1, this study expected that: for goal-driven consumers, LSEEC platforms are more likely to enhance purchase intention compared to ECILS platforms; for recreational consumers, ECILS platforms are more likely to enhance purchase intentions than LSEEC platforms.

### Participation and design

The study employed a 2 (live streaming platform types: LSEEC platforms vs. ECILS platforms) × 2 (consumer types: recreational consumers VS. goal-driven consumers) between-subjects design where platform types and consumer types were manipulated as between-subjects factors. The study was conducted from July 10 to 11. Participants were 120 college students from Northwest University (42.5% males, 57.5% females). All participants were randomly assigned to one of the four experimental groups.

### Procedure

Participants were informed that this study was intended to investigate consumers’ mental simulation of consumption situations. Firstly, participants were asked to choose a platform. In the group of LSEEC platforms, subjects were asked to recall and choose one of their familiar platforms from the LSEEC platforms Taobao and JD; in the group of ECILS platforms, subjects were asked to recall and choose one of their familiar platforms from the ECILS platforms Tik Tok and Kwai. Secondly, participants would read a guiding phrase which manipulated consumer types: “Suppose you are planning to buy a neutral pen and have certain requirements for the features and price of the pen. You use the platform of your choice above and find a live streaming studio selling neutral pens. In order to complete the purchase plan, you enter the studio” (for goal-driven consumers) or “Suppose you do not have any plans to buy a pen, and for pleasure you open the platform of your choice. You find a live streaming room selling neutral pens, and you enter the room out of curiosity” (for recreational consumers). Thirdly, they saw a picture of the product with descriptions blow it (the product descriptions were taken from both Taobao and Tik Tok live broadcasters). Two pictures showed the same neutral pen, but its description highlighted either the design of raw materials (the LSEEC platforms condition) or the feeling of use (the ECILS platforms condition).

To measure purchase intention, we asked participants to indicate their feelings toward the pen used by responding to item taken from Kamins and Gupta (1994) purchase intention scale. As for manipulation checks, the participants answered questions about the consumer types, which were derived from Chen et al.’s (Shen and Wang, 2002; Chen and Dong, 2017) definitions of goal-driven consumers and recreational consumers. Finally, the participants completed their personal information.

### Results

#### Manipulation checks

A one-way analysis of variance (ANOVA) was used to analyse the data. This study’s consumer types (goal-driven vs. recreational) design was effective: the goal-driven consumers had a higher target attribute than the recreational consumers (M goal-driven =5.189，SD = 0.760，*M* recreational =2.783，SD = 0.625; *F*(1, 118) =358.418, *p* < 0.001), and the recreational consumers had a higher recreation attribute than the goal-driven consumers (*M* recreational = 5.139, SD = 0.580, *M* goal-driven =3.056, SD = 0.734; *F*(1, 118) = 297.432，*p* < 0.001).

#### Main effects analysis

This paper used a two-factor ANOVA with purchase intention as the dependent variable. The results showed that there was no significant main effect of platform types on purchase intention [*F*(1, 116) = 0.057, *p* > 0.1]; there was also no significant main effect of consumer types on purchase intention [F(1, 116) = 0.910, *p* > 0.1].

#### Interaction effect of live streaming platform types and consumer types

**Figure 2:**
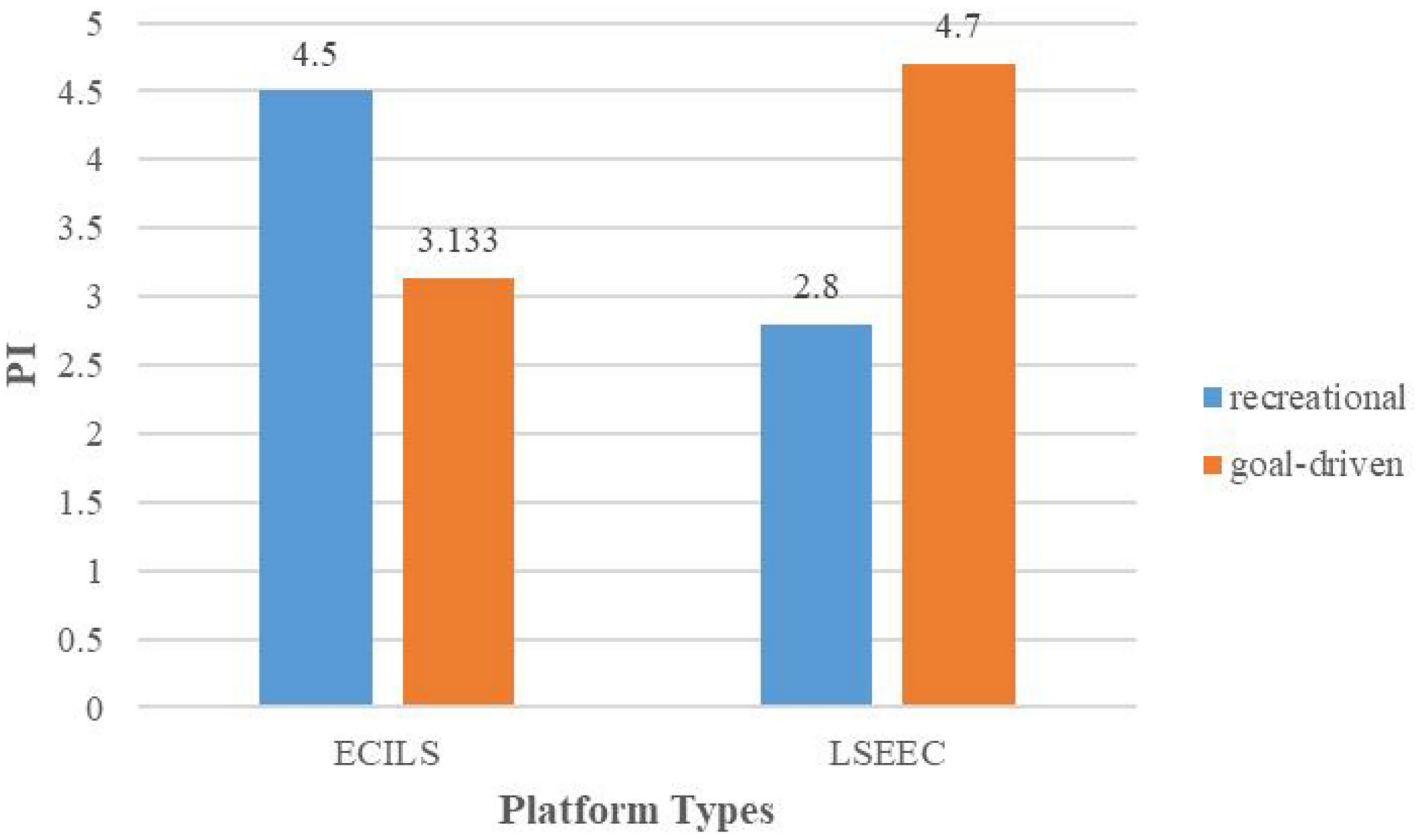
The impact of the interaction between platform types and consumer types on purchase intention.

To verify the effect of the interaction between live streaming shopping platform types and consumer types on purchase intention, this study examined platform types and consumer types as fixed factors with purchase intention as the dependent variable. And the detailed results are shown in Table 1. The results showed that the interaction effect of platform types and consumer types on purchase intention was significant [*F*(1, 116) = 34.123, *p* = 0.000]. Thus, H1 is accepted. The interaction between the two is shown in Figure 2.

**Table 1:** The impact of the interaction between platform type and consumer type on purchase intention.

Source	Type III sum of squares	df	Mean square	*F*	Sig.
Corrected Model	82.300a	3	27.433	11.697	0.000
Intercept	1717.633	1	1717.633	732.341	0.000
platform types	0.133	1	0.133	0.057	0.812
consumer types	2.133	1	2.633	0.910	0.342
platform types *consumer types	80.033	1	80.033	34.123	0.000
Error	272.067	116	2.345		
Total	2072.000	120			
Corrected Total	354.367	119			

a. R Squared = 0.232 (Adjusted R Squared = 0.212).

## Study 2: Mediating role of thinking patterns

Study 2 adopted questionnaires to further verify the interaction effect of platform types and consumer types on purchase intention and to explore the mediating role of thinking patterns.

### Method

We released the questionnaires online in August, 2022. And 298 were returned. The valid questionnaires were 275. Among them, 57.83% were female and 42.17% were male.

The respondents first recalled their live shopping experience and chose one of their commonly used platforms from the LSEEC platforms (Taobao, JD) or ECILS platforms (Tik Tok, Kwai). According to different choices, the respondents received different descriptions. If the respondents chose the LSEEC platform, they were presented with “A host on Taobao/JD live streaming is introducing this product. This is the new T brand body wash. It is rich in medicinal laminaria extract, hyaluronic acid extract, amino acids, citric acid and other ingredients. It has multiple functions such as tightening pores, controlling and reducing skin cortex, penetrating into the basement to clean, and maintaining skin acid–base balance. The product has passed authoritative quality control and will not cause irritation to the body. It’s suitable for babies and pregnant women. This body wash sells for 35 yuan. Hurry up and place your order to buy it!” If the respondents chose the ECILS platform, they would see “an anchor introducing the product in the Tik Tok/Kwai live streaming room. This is a new type of T brand body wash. It is rich in a variety of ingredients that are beneficial to human body. By using it, you can make your skin smooth and obtain super tight skin. The texture of this body wash is absolutely reliable and will not cause any harm to the human body. You can take it home for only 35 yuan. It’s the very time to buy it!.”

After reading the above material, the respondents answered the same questions about consumer types (Shen and Wang, 2002; Chen and Dong, 2017) and purchase intention (Kamins and Gupta, 1994) as in Study 1. In addition, the respondents were also asked to report their thinking patterns by answering the scale of Huang et al. (Novak et al., 2009; Huang and Wang, 2019). The measures were all on a 7-point scale (1 = strongly disagree, 7 = strongly agree). Finally, the respondents also reported their personal information.

### Results

#### Confidence test

To ensure consistency in the results of each measure in the scale measured on the construct to which it belongs, this study used the value of Cronbach′s α coefficient as a criterion for evaluating the reliability of the questionnaire. The results showed that the Cronbach′s α coefficients of the variables all exceed 0.7, indicating that the scale had a high level of reliability for each variable.

#### Validity test

In order to ensure the validity of the questionnaire, the AVE was used to measure the convergent validity of the scale. The results showed that the AVE values of the variables were all greater than 0.5, indicating the scale had good convergence validity. Secondly, the validity of discrimination was evaluated by comparing the square root of the average extracted variance with the absolute value of the correlation coefficient. The results showed that the square root of the mean extracted variance of all variables was greater than the correlation coefficients between the variables and the other variables, reflecting the good discriminant validity of the scale. Finally, the construct validity of the scale is evaluated by testing the factor loadings of variables. The results showed that the factor loading of each item conforms to the standard, reflecting the good construct validity of the scale.

#### Regression analysis

In this paper, SPSS 24.0 was used to conduct regression test on the model (Table 2). Model 1 and Model 2 showed that in the regression with purchase intention, platform types (*β* = 0.150, *p* > 0.05) and consumer types (*β* = 0.107, *p* > 0.05) were not significant. Model 3 showed that the coefficient of interaction term (platform types ×consumer types) was significant in the regression with purchase intention. Thus, H1 is verified.

**Table 2:** Results of regression analysis.

Variable name	Purchase intention		
	Model 1	Model 2	Model 3
Platform types	0.150		
Consumer types		0.107	
Platform types * Consumer types			0.410***
*R* Square	0.023	0.012	0.168
Adjusted *R* Square	0.019	0.008	0.165
*F*	6.289	3.187	55.294***

***means of 1% significance levels.

#### Mediating role of thinking patterns

This study referred to the Bootstrap mediation test and was based on the mediation analysis model proposed by Preacher and Hayes (Preacher et al., 2007) to examine the role of thinking patterns in the interaction between platform types (LSEEC = 1, ECILS = 0) and consumer types (goal-driven consumer = 1, recreational consumer = 0) on purchase intention. We set the sample size to 5,000, the confidence interval to 95% and chose Model 8 for the analysis.

**Figure 3:**
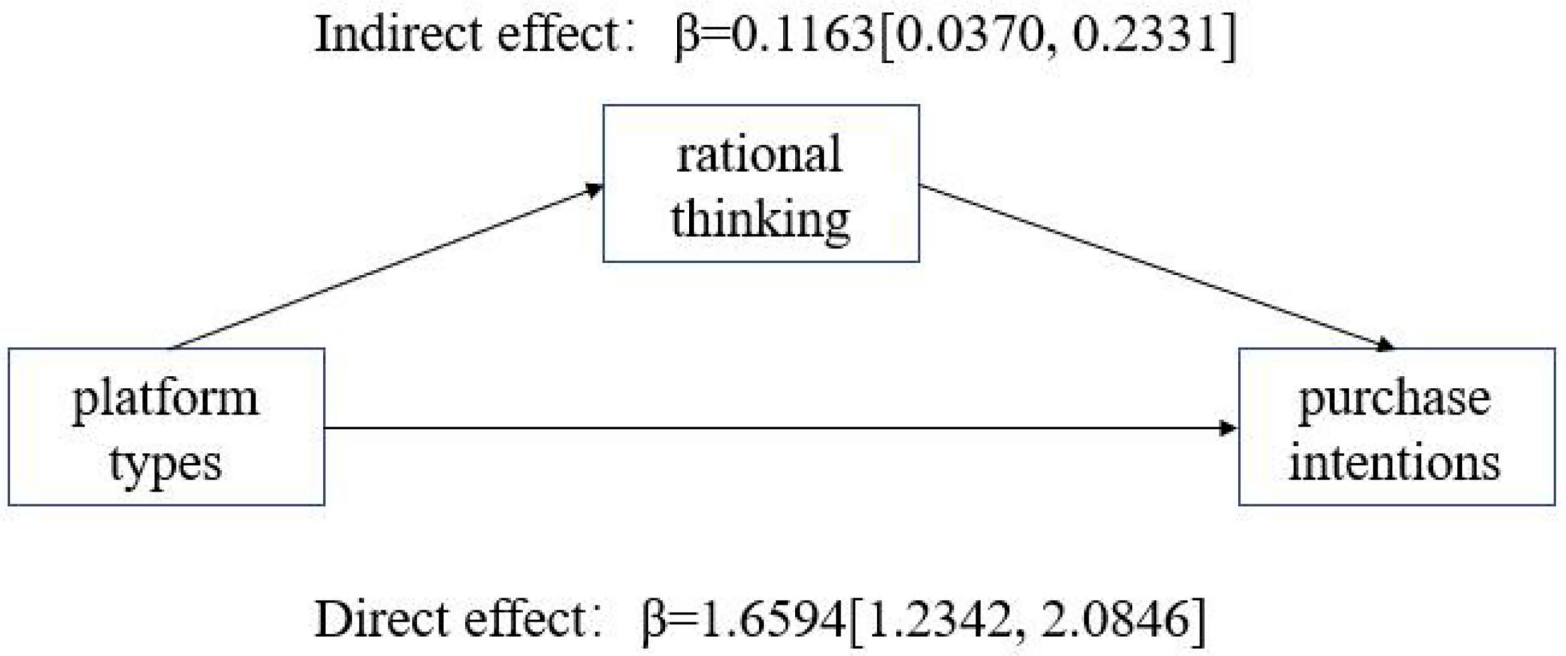
Mediated test of rational thinking (goal-driven consumers).

**Figure 4:**
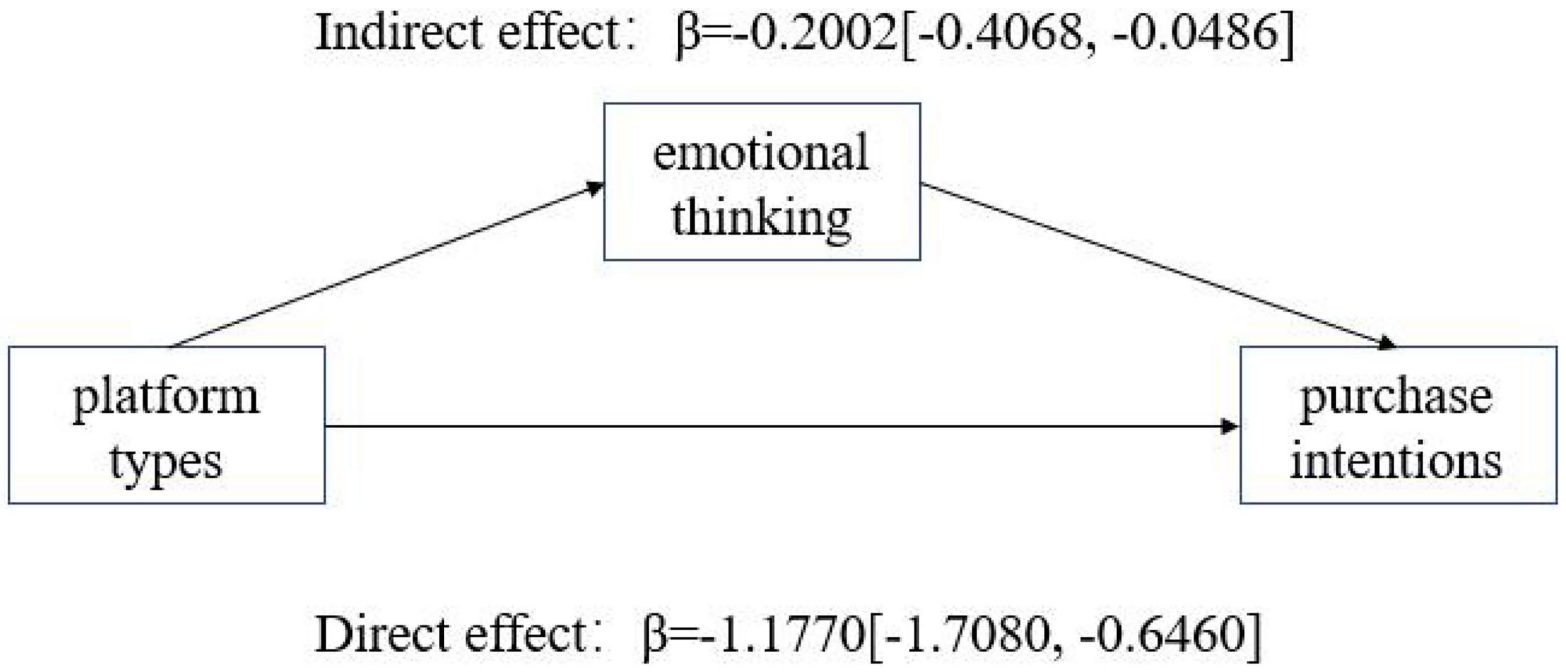
Mediated test of emotional thinking (recreational consumers).

When the consumer type was goal-driven, the indirect effect of rational thinking was LLCL = 0.0370, ULCL = 0.2331 (Figure 3) with an interval not containing 0, indicating a significant indirect effect: for goal-driven consumers, rational thinking mediated the effect, while emotional thinking did not (LLCL = -0.0948, ULCL = 0.1220). When the consumer type was recreational, the indirect effect of emotional thinking was LLCL = −0.4068, ULCL = −0.0486 (Figure 4) and the interval did not contain 0, indicating that the indirect effect was significant: for recreational consumers, emotional thinking mediated the effect, while rational thinking was not significant (LLCL = −0.2169, ULCL = 0.1139). In summary, hypotheses H2a and H2b are tested.

## Discussion and conclusion

This study conducted an experiment and a large-scale online questionnaire to verify the interaction between platform types and consumer types on purchase intention. It also proved the mediating role of thinking patterns in the interaction.

Study 1 indicates that there is no significant effect of the platform types or consumer types on consumers’ purchase intention, but the interaction of platform types and consumer types has a significant effect on purchase intention: for goal-driven consumers, their purchase intention is higher on LSEEC platforms than on ECILS platforms; for recreational consumers, their purchase intention is higher on ECILS platforms than on LSEEC platforms. The current results are consistent with previous studies, which suggested that different live streaming platforms have different characteristics and advantages (Haimson and Tang, 2017; Lu and Chen, 2021). Meanwhile, the current results verify Lu′s conjecture (Lu and Chen, 2021) that platform types might affect consumers’ purchase intention. Besides, previous studies have reported the impact of the anchor (Ma, 2021), audience (Gao et al., 2021), and products (Chen et al., 2022) on consumers in live streaming. The factor employed in the current study is the live streaming platforms. In the current study, LSEEC platforms have stronger commodity attributes and ECILS platforms have stronger social entertainment attributes, which enable them to attract different consumers. In other words, the platform can also be used as a signal for market research, which is different from previous studies that used unconditional money back guarantee (Lee et al., 2005), brand and advertisement (Kirmani, 1990), and online Word of Mouth (C.M.K. Cheung et al., 2014) as market signals.

The results of study 2 show the same interaction effect as study 1. Meanwhile, Study 2 also verifies the mediating mechanism of thinking patterns. The results show that goal-driven consumers’ purchase intention is enhanced through rational thinking, while recreational consumers’ purchase intention is enhanced through emotional thinking. All of these results support the primary hypothesis. As reviewed in the theoretical background and hypotheses section, several studies have consistently suggested that consumers’ thinking would be affected by situational factors (Novak et al., 2009; Samson and Voyer, 2014; Huang and Wang, 2019; He et al., 2021), and different characteristics of the platform may cause different thinking patterns (Erdogan, 1999; Raghunathan and Corfman, 2006). Thus, the current results prove the rationality of predecessor’s research.

In conclusion, platform types and consumer types have an interaction effect on purchase intention in live streaming shopping; thinking patterns play a mediating role between them: for goal-driven consumers, LSEEC platforms are more likely to stimulate their rational thinking and enhance their purchase intention; for recreational consumers, ECILS platforms are more likely to stimulate their emotional thinking and enhance their purchase intention.

### Implications and contribution

#### Theoretical contributions

There is already some literature exploring the classification and characteristics of live streaming platforms. For example, some studies have divided live streaming platforms into traditional e-commerce platforms and content e-commerce platforms (Zhu and Chen, 2016; China Consumers’ Association, 2020; Zang and Zhao, 2021), while others have divided live streaming platforms into social e-commerce platforms, content e-commerce platforms, video content e-commerce platforms, etc. (IResearch, 2020). However, although these studies pointed out the characteristics of each type, they were limited to descriptive exploration of characteristics and classification. While this study explores the relationship between platform types and consumers’ purchase intentions based on previous classifications of platforms, extending the depth of research related to live streaming platforms.

Previous studies have found differences in consumer thinking patterns depending on the shopping scenario, such as shopping online versus offline (Lynch and Ariely, 2000; Rosen and Howard, 2000; Chen and Tian, 2018), shopping using mobile versus shopping using PC (Dijksterhuis and Olden, 2006; Brasel and Gips, 2014; Huang and Wang, 2019), etc. This study focuses on the live streaming platform scenario and finds that the type of platform and consumers jointly affect the thinking patterns of live streaming consumers, which enriches the research findings related to shopping thinking in live streaming shopping scenario.

Unlike previous online shopping studies that focused on two variables, consumer types and purchase intention (Yang et al., 2017; Tao and You, 2021; Wang et al., 2021), this study innovatively combines consumer types and platform types, to explore the interaction effect on purchase intention, and to find the mediating role of thinking patterns. The results reveal the important role of thinking patterns on purchase intention, and suggest complex antecedents of thinking patterns in live shopping decisions.

#### Practical implications

More importantly, the findings of this study bring some practical insights into live streaming marketing: firstly, it helps companies to develop a further understanding of the platform types. In practice, many companies run live streaming in official flagship shops on LSEEC platforms, and use the shopping attributes of the platforms to attract consumption. However, this study finds that there are boundary conditions for the role of these types of platforms. LSEEC platforms are more likely to enhance the purchase intentions of goal-driven consumers, but do not significantly contribute to the formation of purchase intentions of recreational consumers. On the contrary, ECILS platforms can attract a large number of recreational consumers by leveraging the fan base of the platforms.

Secondly, it helps enterprises to establish a suitable live streaming platform strategy. Companies should accurately analyze the characteristics of their target consumers to choose the right platform or to build an efficient live streaming matrix for their products. For example, basic introductory products often focus on goal-driven consumers and the live sales should therefore be carried out on LSEEC platforms; the co-branded products often focus on recreational consumers and the live sales should therefore be carried out on ECILS platforms.

In addition, it helps companies to plan for effective content on live streaming. This study finds that LSEEC platforms are more likely to motivate goal-driven consumers due to its objective, professional and detailed product information that fits their thinking patterns. Companies should reinforce this feature and make full use of the professional shopping attributes of LSEEC platforms. Conversely, recreational consumers are more likely to prefer live streaming rooms on ECILS platforms. So, companies should open live streaming rooms on such platforms differently from LSEEC platforms: highlighting entertainment and fun, using non-product factors to attract consumers, and taking advantage of the fan base.

#### Limitations and further study direction

This paper explores the effect of the interaction between platform types and consumer types on purchase intentions and provides an in-depth analysis of the underlying influence mechanisms, but there are certain limitations and directions for future related research. Firstly, this paper conducts research through manipulated experiments and large-scale questionnaires. Future exploration can try to explore the interaction effect of the two at the level of consumer behavioral outcomes by using real data from the platforms. Secondly, this paper explores the effect of the interaction between platform types and consumer types on purchase intention, but there are other outcome variables in live shopping, such as customer engagement, customer word-of-mouth and brand loyalty, which future research could enrich to explore the outcome variables. Finally, this paper explores the impact of different platform types on consumers from a thinking patterns perspective, but the differences between LSEEC platforms and ECILS platforms are not limited to this point, and the impact of other mediating mechanisms could be considered in the future.

## Data availability statement

The raw data supporting the conclusions of this article will be made available by the authors, without undue reservation.

## Ethics statement

The studies involving human participants were reviewed and approved by the School of Economics and Management, Northwest University. Written informed consent for participation was not required for this study in accordance with the national legislation and the institutional requirements.

## Author contributions

YX: conceptualization, project administration, supervision, writing—original draft, writing—review and editing, and funding acquisition. KD: conceptualization, formal analysis, investigation, data curation, writing—original draft, writing—review and editing, and visualization. PG: conceptualization, project administration, supervision, and funding acquisition. All authors contributed to the article and approved the submitted version.

## Funding

This work was supported by grant from the National Natural Science Foundation of China (71972156 and 71802158), Shaanxi Social Science Fund (2018S42), Shaanxi Natural Science Fund (2014JM9370; 2020JQ-608).

## Conflict of interest

The authors declare that the research was conducted in the absence of any commercial or financial relationships that could be construed as a potential conflict of interest.

## Publisher’s note

All claims expressed in this article are solely those of the authors and do not necessarily represent those of their affiliated organizations, or those of the publisher, the editors and the reviewers. Any product that may be evaluated in this article, or claim that may be made by its manufacturer, is not guaranteed or endorsed by the publisher.

## References

[ref1] Brasel S. A. Gips J. (2014). Tablets, touchscreens, and touchpads: how varying touch interfaces trigger psychological ownership and endowment. J. Consum. Psychol. 24, 226–233. doi: 10.1016/j.jcps.2013.10.003

[ref2] Cai J. Wohn D. Y. Mittal A. Sureshbabu D. (2018). Utilitarian and hedonic motivations for live streaming shopping. Proceedings of the 2018 ACM International Conference on Interactive Experiences for TV and Online Video, June 2018

[ref3] Chen H. Chen H. Tian X. (2022). The dual-process model of product information and habit in influencing consumers’ purchase intention: the role of live streaming features. Electron. Commer. Res. Applicat. 53:101150. doi: 10.1016/j.elerap.2022.101150

[ref4] Chen M. Dong P. (2017). Characteristics of online consumer behaviors. China Bus. Mark. 31, 80–85. doi: 10.14089/j.cnki.cn11-3664/f.2017.02.010

[ref5] Chen L. Tian X. (2018). Research on the influential factors and mechanism of consumer channel migration between online and offline. China Bus Mark. 32, 75–83. doi: 10.14089/j.cnki.cn11-3664/f.2018.05.008

[ref6] Chen H. Zhang S. Shao B. Gao W. Xu Y. (2020). How do interpersonal interaction factors affect buyers’ purchase intention in live stream shopping? The mediating effects of swift guanxi. Internet Res. 32, 335–361. doi: 10.1108/intr-05-2020-0252

[ref7] Cheung C. M. K. Xiao B. S. Liu I. L. B. (2014). Do actions speak louder than voices? The signaling role of social information cues in influencing consumer purchase decisions. Decis. Support. Syst. 65, 50–58. doi: 10.1016/j.dss.2014.05.002

[ref8] China Consumers’ Association. (2020). Live e-commerce shopping consumer satisfaction online survey report. Available at: https://www.cca.org.cn/jmxf/detail/29533.html (Accessed August 10, 2022)

[ref9] Del Missier F. Mäntylä T. de Bruin W. B. (2012). Decision-making competence, executive functioning, and general cognitive abilities. J. Behav. Decis. Making 25, 331–351. doi: 10.1002/bdm.731

[ref10] Dijksterhuis A. Olden Z. V. (2006). On the benefits of thinking unconsciously: unconscious thought can increase post-choice satisfaction. JESP 42, 627–631. doi: 10.1016/j.jesp.2005.10.008

[ref13] Erdogan (1999). Celebrity endorsement: A literature review. JMM. 15, 291–314. doi: 10.1362/026725799784870379

[ref14] Evans J. S. (2008). Dual-processing accounts of reasoning, judgment, and social cognition. Annu. Rev. Psychol. 59, 255–278. doi: 10.1146/annurev.psych.59.103006.093629, PMID: 18154502

[ref15] Gao L. Wang H. Zhang Z. Zhuang H. Zhou B. (2022). HetInf: social influence prediction with heterogeneous graph neural network. Front. Phys. 9:787185. doi: 10.3389/fphy.2021.787185

[ref16] Gao X. Xu X. Tayyab S. M. U. Li Q. (2021). How the live streaming commerce viewers process the persuasive message: an ELM perspective and the moderating effect of mindfulness. Electron. Commer. Res. Applicat. 49:101087. doi: 10.1016/j.elerap.2021.101087

[ref17] Guo L. Hu X. Lu J. Ma L. (2021). Effects of customer trust on engagement in live streaming commerce: mediating role of swift guanxi. Internet Res. 31, 1718–1744. doi: 10.1108/intr-02-2020-0078

[ref18] Haimson O. L. Tang J. C. (2017). What makes live events engaging on facebook live,periscope, and snapchat. Proceedings of the 2017 CHI Conference on Human Factors in Computing Systems – CHI ‘17, New York: ACM 48–60.

[ref19] Hao Y. (2010). An empirical study on the influence of online comments on consumer perception and purchasing behavior. dissertation. Harbin: Harbin Institute of Technology.

[ref20] He R. Li B. Zhang S. Cui X. Lei L. (2021). The effect of time and money concepts on consumers’ purchase decision and its psychological mechanism. Adv. Psychol. Sci. 29, 1684–1695. doi: 10.3724/SP.J.1042.2021.01684

[ref21] Ho C. Liu Y. Chen M. (2022). Factors influencing watching and purchase intentions on live streaming platforms: from a 7Ps marketing mix perspective. Information 13:239. doi: 10.3390/info13050239

[ref22] Hu M. Chaudhry S. S. (2020). Enhancing consumer engagement in e-commerce live streaming via relational bonds. Internet Res. 30, 1019–1041. doi: 10.1108/INTR-03-2019-0082

[ref23] Huang M. Wang W. (2019). Does mobile shopping make fast decisions? The role of contextual factors and thinking style. Acta Psychol. Sin. 51, 612–624. doi: 10.3724/SP.J.1041.2019.00612

[ref24] Huang M. Ye Q. Wang W. (2021). The interaction effect of broadcaster and product type on consumers’ purchase intention and behaviors in livestreaming shopping. Nankai Bus. Rev. 25, 1–21. doi: 10.3969/j.issn.1008-3448.2022.06.001

[ref25] iiMedia Research. (2020a). H1 2020 China live e-commerce platform ecological chain layout and typical case data analysis report. Available at: https://report.iimedia.cn/repo1-0/39071.html (Accessed August 10, 2022).

[ref26] iiMedia Research. (2020b). China’s live broadcast e-commerce industry data, development summary and trend analysis from November to December. Available at: https://www.iimedia.cn/c1020/76746.html (Accessed August 11, 2022).

[ref27] IResearch. (2020). Chinese video content e-commerce industry white paper in 2020. Available at: https://baijiahao.baidu.com/s?id=1679035818588407744&wfr=spider&for=pc (Accessed August 10, 2022).

[ref28] Kamins M. A. Gupta K. (1994). Congruence between spokesperson and product type: a matchup hypothesis perspective. Psychol. Market. 11, 569–586. doi: 10.1002/mar.4220110605

[ref29] Kirmani A. (1990). The effect of perceived advertising costs on brand perceptions. JCR 17:160. doi: 10.1086/208546

[ref30] Lee B. Ang L. Dubelaar C. (2005). Lemons on the web: a signalling approach to the problem of trust in internet commerce. J. Econ. Psychol. 26, 607–623. doi: 10.1016/j.joep.2005.01.001

[ref31] Li X. Qitao Z. Zhang W. Shi M. Liu B. (2021). Study on the factors influencing users’ purchase intention on live-streaming e-commerce platforms: evidence from the live-streaming platform of TikTok. J. China Stud. 24, 25–49. doi: 10.20288/jcs.2021.24.3.25

[ref32] Liu W. (2020). Research on the marketing strategy of Taobao live broadcast of publishing institutions under the new media perspective. China Publish. J. 07, 19–22. doi: 10.3969/j.issn.1002-4166.2020.07.006

[ref33] Liu Z. Zhao X. Long W. (2020). The formation mechanism of Consumers’ purchase intention under the influencer marketing–an analysis based on grounded theory. China Bus. Market. 34, 48–57. doi: 10.14089/j.cnki.cn11-3664/f.2020.08.005

[ref34] Lu B. Chen Z. (2021). Live streaming commerce and consumers’ purchase intention: an uncertainty reduction perspective. Inform. Manag. 58:103509. doi: 10.1016/j.im.2021.103509

[ref35] Luo H. Cheng S. Zhou W. (2021). The factors influencing sales in online celebrities’ live streaming. In 2021 IEEE International Conference on Information Communication and Software Engineering

[ref36] Lynch J. G. Ariely D. (2000). Wine online:search costs affect competition on price，quality，and distribution. Market. Sci. 19, 83–103. doi: 10.1287/mksc.19.1.83.15183

[ref37] Ma Y. (2021). To shop or not: understanding Chinese consumers’ live-stream shopping intentions from the perspectives of uses and gratifications, perceived network size, perceptions of digital celebrities, and shopping orientations. Telemat. Inform. 59:101562. doi: 10.1016/j.tele.2021.101562

[ref38] Ma S. Wang Y. (2015). Delight the customer or solve their problems in virtual brand communities? Empirical investigation of the differentiated impacts of commitment. Econ. Manag. 37, 77–86. doi: 10.19616/j.cnki.bmj.2015.01.010

[ref39] Ma Q. Zhao J. Zhang Y. Hao J. (2012). A research on the formation mechanism of Customers’ initial trust under C2C environment: the moderation effect of online shopping experience. Bus. Rev. 24, 70–81. doi: 10.14120/j.cnki.cn11-5057/f.2012.07.006

[ref40] Novak T. P. Hoffman D. L. John D. S. A. E. (2009). The fit of thinking style and situation: new measures of situation-specific experiential and rational cognition. JCR 36, 56–72. doi: 10.1086/596026

[ref41] Preacher K. J. Rucker D. D. Hayes A. F. (2007). Addressing moderated mediation hypotheses: theory, methods, and prescriptions. Multivar. Behav. Res. 42, 185–227. doi: 10.1080/00273170701341316, PMID: 26821081

[ref42] Raghunathan R. Corfman K. (2006). Is happiness shared doubled and sadness shared halved? Social influence on enjoyment of hedonic experiences. JMR 43, 386–394. doi: 10.1509/jmkr.43.3.386

[ref43] Rosen K. T. Howard A. L. (2000). E-retail: gold rush or fool’s gold? CMR 42, 72–100. doi: 10.2307/41166043

[ref44] Rungruangjit W. (2022). What drives Taobao live streaming commerce? The role of parasocial relationships, congruence and source credibility in Chinese consumers’ purchase intentions. Heliyon 8:e9676. doi: 10.1016/j.heliyon.2022.e09676, PMID: 35756134 PMC9213714

[ref45] Samson A. Voyer B. G. (2014). Emergency purchasing situations: implications for consumer decision-making. J. Econ. Psychol. 44, 21–33. doi: 10.1016/j.joep.2014.05.004

[ref46] Shen W. Wang C. (2002). Analysis of online customers’ purchasing behavior. Commerc. Econom. Rev. 3, 56–58. doi: 10.3969/j.issn.1008-2778.2002.04.003

[ref47] Sloman S. A. (1996). The empirical case for two systems of reasoning. Psychol. Bull. 119, 3–22. doi: 10.1037/0033-2909.119.1.3

[ref48] Strack F. Werth L. Deutsch R. (2006). Reflective and impulsive determinants of consumer behavior. J. Consum. Psychol. 16, 205–216. doi: 10.1207/s15327663jcp1603_2

[ref49] Sun Y. Shao X. Li X. Guo Y. Nie K. (2019). How live streaming influences purchase intentions in social commerce: an IT affordance perspective. Electron. Commer. Rev. Appl. 37:100886. doi: 10.1016/j.elerap.2019.100886

[ref50] Tao L. You T. (2021). Method for ranking hotels based on online ratings and considering consumer types. Operat. Res. Manag. Sci. 30, 122–128. doi: 10.12005/orms.2021.0154

[ref51] Wang P. (2015). Exploring the influence of electronic word-of-mouth on tourists’ visit intention a dual process approach. J. Syst. Inf. Technol. 17, 381–395. doi: 10.1108/JSIT-04-2015-0027

[ref52] Wang B. (2021). The essence, logic and trend of livestreaming e-commerce. China Bus. Market. 35, 48–57. doi: 10.14089/j.cnki.cn11-3664/f.2021.04.005

[ref53] Wang F. Jiang J. (2021). How does the internet short video business model realize value creation? A comparative case study of Douyin and Kuaishou. Fore Econ. Manag. 43, 3–19. doi: 10.16538/j.cnki.fem.20210103.101

[ref54] Wang L. Zhang Z. (2021). Analysis on the high sales anchor marketing of books live broadcast with goods–an empirical study on book live broadcast account based on Tiktok app. China Publish. J. 11, 19–25. doi: 10.3969/j.issn.1002-4166.2021.11.005

[ref55] Wang H. Zhou S. Li S. Ke G. (2021). Research on improving the effect of touch screen purchase advertising – evidence from APP experiments. J. Manag. Sci. 34, 142–154. doi: 10.3969/j.issn.1672-0334.2021.06.014

[ref56] Yang Y. Liao X. Liu M. (2017). An empirical analysis of the relationship between purchase intention and purchase probability of online consumers—based on hedonism and utilitarianism. Proc. Bus. Econ. Stud. 7, 49–52. doi: 10.3969/j.issn.1002-5863.2017.07.018

[ref57] Yang J. Wang L. (2021). Factors influencing consumer repurchase intention in livestreaming E-commerce. China Bus. Market. 35, 56–66. doi: 10.14089/j.cnki.cn11-3664/f.2021.11.006

[ref58] Zang C. Zhao T. (2021). The development status, problems and regulation path of e-commerce live streaming in China. New Media Res. 7, 49–53. doi: 10.16604/j.cnki.issn2096-0360.2021.10.014

[ref59] Zhang M. Hai Y. T. Song Y. (2022). The influence of host characteristics on consumers’ purchase intention to buy tea products in live streaming-focused on the difference between ordinary celebrity and public officers on Tik Tok platform. Korean Chin. Soc. Sci. Stud. 63, 261–287. doi: 10.36527/kcsss.20.2.13

[ref60] Zhang K. Z. Zhao S. J. Cheung C. M. Lee M. K. (2014). Examining the influence of online reviews on consumers’ decision-making: a heuristic–systematic model. Decis. Support. Syst. 67, 78–89. doi: 10.1016/j.dss.2014.08.005

[ref61] Zhao D. (2018). Research on brand exposure and sales transformation in the era of online live broadcasting-take shopping live broadcast platform as an example. Proc. Bus. Econ. Stud. 01, 62–64. doi: 10.3969/j.issn.1002-5863.2018.01.019

[ref62] Zhu X. Chen J. (2016). A review of the research on social e-commerce in China. J. Mod. Informat. 36, 172–177. doi: 10.3969/j.issn.1008-0821.2016.01.030

[ref63] Zhu Y. Zhu Y. (2022). The Research of Multi-Dimensional Relation in Digital Commerce: Based the Interpretation of Online Live Shopping. Econ. Manag. 36, 19–25. doi: 10.3969/j.issn.1003-3890.2022.01.004

[ref64] Zhu C. Wang J. Ji Y. (2004). Research on consumer personality and consumer behavior. Inq. Econ. 12, 118–122. doi: 10.3969/j.issn.1006-2912.2004.12.037

